# Genetic diversity of Olive flounder (*Paralichthys olivaceus*) and the impact of selective breeding on Korean populations

**DOI:** 10.1371/journal.pone.0318672

**Published:** 2025-04-16

**Authors:** Euiseo Hong, Hyun-Chul Kim, Jeong-Ho Lee, Woonyoung Jeong, Phuong Thanh N. Dinh, Waruni Ekanayake, Jong-Won Park, Minhwan Jeong, Dain Lee, Julan Kim, Yoonsik Kim, Seung Hwan Lee, Yoonji Chung

**Affiliations:** 1 Department of Bio-Big Data and Precision Agriculture, Chungnam National University, Daejeon, Republic of Korea; 2 Genetics and Breeding Research Center, National Institute of Fisheries Science, Geoje, Republic of Korea; 3 Department of Bio-AI Convergence, Chungnam National University, Daejeon, Republic of Korea; 4 Division of Animal & Dairy Science, Chungnam National University, Daejeon, Republic of Korea; 5 Institute of Agricultural Science, Chungnam National University, Daejeon, Republic of Korea; Sher-e-Kashmir University of Agricultural Sciences and Technology of Kashmir, INDIA

## Abstract

This study aimed to identify the population structure and genetic diversity of olive flounder (*Paralichthys olivaceus*) in Korea and to examine the potential for genetic improvement in aquaculture populations. PCA showed NIFS and FarmA as closely related clusters, while FarmB exhibited moderate differentiation with greater variability. Fst analysis indicated high similarity between NIFS and farmed populations (0.021–0.043) but significant differentiation from wild populations (0.274–0.295). Admixture analysis highlighted a shared ancestral component (over 70%) among NIFS and farmed populations, contrasting with the unique genetic makeup of wild populations. The phylogenetic tree confirmed these patterns, with NIFS and FarmA forming close branches, FarmB showing intermediate placement, and wild populations clustering separately. Additionally, genomic estimated breeding values for body weight showed no significant differences between FarmA and FarmB, while prediction accuracy was higher for FarmA (47%) compared to FarmB (45%), indicating a closer genetic relationship between NIFS and FarmA. These findings emphasize the critical role of selective breeding and gene flow in shaping the genetic structure of farmed populations, offering valuable insights for improving growth traits and maintaining genetic diversity in aquaculture.

## Introduction

Olive flounder is a prominent species of flatfish renowned for its delicate flavor, and nutritional value, making it a sought-after species in the global seafood industry. The species is widely known for its high growth rate, resistance to disease, feed efficiency, and tolerance to water temperature changes, thus, well suited for aquaculture production [1,2]. Originating from the coastal waters of East Asia, South Korea and Japan are currently the leading producers of commercially produced olive flounder, while China also contributes to a considerable proportion of the world’s total production [3]. In South Korea, large-scale commercial production of olive flounder was initiated in the 1990s [4] and since then it has been considered one of the country’s major marine fin fish breeds. In 2020, the country produced 43,813 metric tons of olive flounder which accounted for 49% of the national aquaculture production and 46% of the international olive flounder production [5]. A larger proportion of aquacultured olive flounder is consumed domestically while the rest is exported to Japan, the United States, and Taiwan [4].

**Fig 1:**
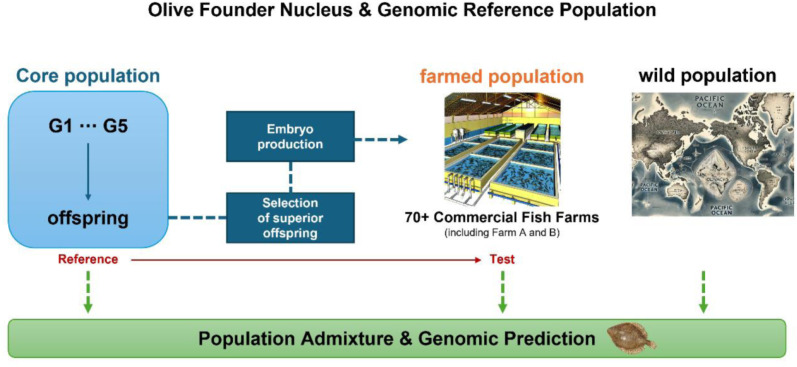
Olive flounder breeding scheme and fertilized eggs distribution system by NIFS. The olive flounder breeding scheme and fertilized eggs distribution system by NIFS are depicted in Fig 1. Between 2010 and 2020, over 450 million fertilized eggs were distributed to more than 70 aquaculture farms across the country. These eggs were derived from the selective breeding of superior broodstock across multiple generations (G1 to G5). This figure was created using OpenAI’s DALL-E platform. The map and illustrations are AI-generated and do not rely on specific geospatial datasets or shape files. Use of this figure is subject to OpenAI’s terms and conditions.

Since 2004, the National Institute of Fisheries Science (NIFS) has initiated a selective breeding program targeting growth performance as the breeding trait. A fast-growing line was industrialized under the name “King Nupchi.” Between 2010 and 2020, more than 450 million fertilized eggs were distributed to over 70 aquaculture farms nationwide (**Fig 1 and** **S1 Note**). These eggs originated from the selective breeding of superior broodstock across multiple generations (G1 to G5). Genetic improvement through selective breeding is cumulative across generations, leading to continuous enhancements in productivity [6]. To secure the prospects of future selective breeding, it is essential to characterize the population structure and maintain genetic diversity [7].

Studies on the population structure and genetic diversity of Korean olive flounder (Paralichthys olivaceus) have been conducted. Kim et al. analyzed the genetic structure of 459 individuals collected from five wild populations and three farmed populations in Korea using nine polymorphic microsatellite (MS) loci. Their findings demonstrated that farmed populations still maintain sufficient genetic diversity [8]. Jung et al. evaluated the genetic diversity of juvenile olive flounders produced from farmed broodstock using seven microsatellite DNA markers. Their study assessed the genetic health of released juveniles and provided recommendations for improving aquaculture programs [9].

Numerous studies have conducted genomic analyses of Korean olive flounder (Paralichthys olivaceus) using microsatellite marker [7–9]. However, there is a lack of research on the population structure and genetic diversity of Korean olive flounder populations, including private aquaculture farms and wild populations, using SNP markers. This study aimed to identify the population structure and genetic diversity of olive flounder in Korea. Admixture analysis, *F*_*st*_, and neighbor-joining tree analyses were performed to assess genetic differentiation between populations. PCA was conducted to evaluate genetic diversity. Additionally, to examine the potential for genetic improvement in aquaculture populations, comparison on genomic breeding values was made between selected breeding populations and aquaculture farms.

## Materials and methods

### Ethics statement

The animal study protocol was approved by the Institutional Animal Care and Use Committee (IACUC) at National Institute of Fisheries Science (Approval number: 2023-NIFS-IACUC-39, date of approval: 17 April 2023). The experimental animals were not anesthetized or euthanized in this study. We confirmed that all methods are reported in accordance with ARRIVE guidelines (https://arriveguidelines.org) for the reporting of animal experiments.

### Animals and phenotypes

Fish samples were collected from three different sources [1], the National Institute of Fisheries Science (NIFS), South Korea [2]; a private source which includes two major aquatic farming facilities which sell fertilized egg of olive flounder: Fish farm A and B [3]; NCBI. The NIFS dataset consisted of 992 samples of *Paralichthys olivaceus* with phenotypes for body weight (BW in g) and total length (TL in cm) and genotypes obtained using Affymetrix 60K SNP chip (S1 Fig). The specifics about fish stocks, family production, and data collection are explained in detail in S1 Note and the previous study [10]. The data from the private source included genotypes of 200 fishes, 100 fishes each from two aquaculture farms, of *Paralichthys olivaceus* obtained using a 60K SNP chip. Finally, the data acquired from NCBI consisted of genotypes of 24 samples of *Paralichthys olivaceus* (NCBI_POL), *Paralichthys adspersus* (NCBI_PAD), and *Paralichthys orbignyanus* (NCBI_POR) obtained using whole-genome sequencing (WGS). NCBI_POL represents the founder population of Korean olive flounder, while NCBI_POR and NCBI_PAD were used to compare the genetic structure of two farms and NIFS. In addition, 302 samples from the 1^st^ to 5^th^ generations of the NIFS population were analyzed to assess genetic similarity with farmed populations and wild populations (NCBI).

### Genotypes

The data preprocessing was carried out in the following procedure. After the raw sequence data from all sources were mapped to the Newly assembled de novo reference genome of Korean olive flounder [11], the variant calling was carried out. Sequence analysis was performed using GATK variant discovery pipeline to call variants. The key steps in the pipeline include read trimming and adapter removal by trimmomatic, read alignment to the Newly assembled de novo reference genome of Korean olive flounder using bwa-mem. In GATK, the ‘read-group’ (RG tag) is essential for subsequent analyses. To assign reads to a new group, the AddOrReplaceReadGroups tool was utilized. To identify duplicate reads and tag them, the MarkDuplicates tool was applied. Since DNA is complementary, there are sequences that begin at the 5’ end and others at the 3’ end. As the analysis involves paired-end reads, the reads originating from the 5’ and 3’ ends are referred to as “mates.” In this context, any discrepancies in mate-pair information were corrected using the FixMateInformation tool. For variant calling, we used GATK HaplotypeCallerSpark in GVCF mode to process the BAM file of each individual and obtained a separate gVCF for each individual. To merge the GVCFs of individual samples prior to joint calling, GenomicsDBImport was used. Then, GenotypeGVCFs was employed to perform joint genotyping on the data stored in the GenomicsDB. After joint calling, the VCF files generated for each chromosome were combined into a single VCF file using GatherVcfs. The VariantFiltration module was then used to set filtering criteria and perform hard-filtering of variants. Finally, the SelectVariants module was used to exclude filtered variants as well as non-variant sites. the NCBI dataset yielded 40,461,711 SNPs. 992 and 200 animals for NIFS and aquaculture farms datasets (farm A and B), respectively were genotyped on 60k arrays (60k SNP, Affymetrix). The number of SNPs on 60K chip is 60,435 SNPs. Next, the SNPs that were mutual in all datasets were selected and a total of 51,506 SNPs were identified as common across all datasets. For the quality control, out of 51,506 common SNPs, the SNPs with a call rate less than 90% and a minor allele frequency (MAF) less than 1% were removed and 50,917 SNPs were retained. Finally, the population structure and diversity analysis and genetic evaluation were implemented with the final set of 50,917 SNPs.

### Population structure and diversity analysis

Population structure and diversity analysis were performed using a comprehensive set of algorithms to assess the genetic variability and relationships among different olive flounder populations.

#### Principal component analysis (PCA).

Principal component analysis (PCA) was implemented to reduce the dimensionality for investigating the population structure within and between populations. Plink1.9 [12] was used for the PCA to obtain eigenvalues and eigenvectors, and the results were visualized using R software to show the genetic distances (genetic similarities and differences) between the individuals from different olive flounder populations in a multidimensional space.

#### Neighbor-joining tree (Phylogenic Tree) and Fst.

To illustrate the phylogenetic relationships among the species, a neighbor-joining tree based on pairwise genetic distances between species was constructed using SNPhylo.20180901 [13]. The pairwise fixation index value (Fst) [14] was performed for genetic differentiation among the populations in plink1.9.

#### Admixture analysis.

The ADMIXTURE v1.23 [15] program was used to detect possible mixtures of ancestral populations, with four ancestral populations (K = 4) to understand the genetic composition of the NIFS, two aquaculture farms, and NCBI populations.

### Genetic evaluation

#### Genetic parameters.

The genetic parameters were estimated for body weight (BW) and total length (TL) for the NIFS dataset through univariate analysis. The analysis was conducted using the genomic best linear unbiased prediction (gBLUP) method based on a reference population of 972 individuals from 1^st^ to 5^th^ generation of NIFS dataset using BLUPF90 v2.57 family of programs [16].

#### Comparison of average breeding value between three NIFS and farmed populations.

The average estimated breeding values of NIFS and two aquacultures were compared to investigate the effect of selective breeding by both NIFS and commercial farms. Genomic estimated breeding values (gEBV) were calculated for BW and TL with the genomic best linear unbiased prediction (gBLUP) method using BLUPF90 v2.57 family of programs [16]. gBLUP uses the genomic relationship matrix (**G**) based on SNP markers. The **G** matrix is expressed as below:


$$G=\frac{MM'}{\sum_{i=1}^{m}2p_{i}\left(1-p_{i}\right)}$$

Then,


$$\text{Var}\left(u\right)=G\sigma_{u}^{2}$$

Where **M** is the genotype matrix, *m* is the total number of SNP markers, and $p_{i}$ is the allele frequency of the $i^{th}$ SNP. The genetic parameters were estimated with 979 animals of NIFS in detail in S2 Note. The gBLUP model used is as follows:


y=Xb+Zu+ε

Where **y** is a vector of observed phenotypes, **X** is a design matrix relating the fixed effects to animals, **b** is a vector of fixed effects, **Z** is a design matrix which allocates the records in **y** to the random effects in **u** is a vector of random effects (estimated breeding values; EBVs), and **ε** is a vector of residual terms.

A random sample of 100 animals from the 7^th^ generation of the NIFS dataset and 100 animals each from farm A and B datasets were used as the test set, and the remaining 872 animals from the NIFS dataset were used as the reference set. Obtained gEBV were then compared between NIFS and commercial farms.

### AI-based figure generation

Fig 1 was generated using OpenAI’s DALL-E model, an artificial intelligence (AI) tool designed to create images based on textual descriptions. We utilized this tool to visually represent the fish farm and wild population groups described in the study. The textual prompts provided to the AI tool were carefully designed to clearly distinguish the characteristics of each group, ensuring that the generated image accurately reflected the study’s concepts and data. The AI-generated output was reviewed and validated to confirm its suitability and alignment with the study’s objectives. No pre-existing datasets, shapefiles, or GIS data were used in the creation of this image; instead, it was entirely produced through AI-based visualization techniques. We take full responsibility for ensuring the reliability of the AI-generated content and verifying its consistency with the study results. The use of this AI tool did not influence the interpretation, results, or conclusions of the study and was employed solely as a supplementary visual aid to support the presentation of the research.

## Results

### Population structure and diversity

#### Principal Component analysis (PCA).

**Fig 2:**
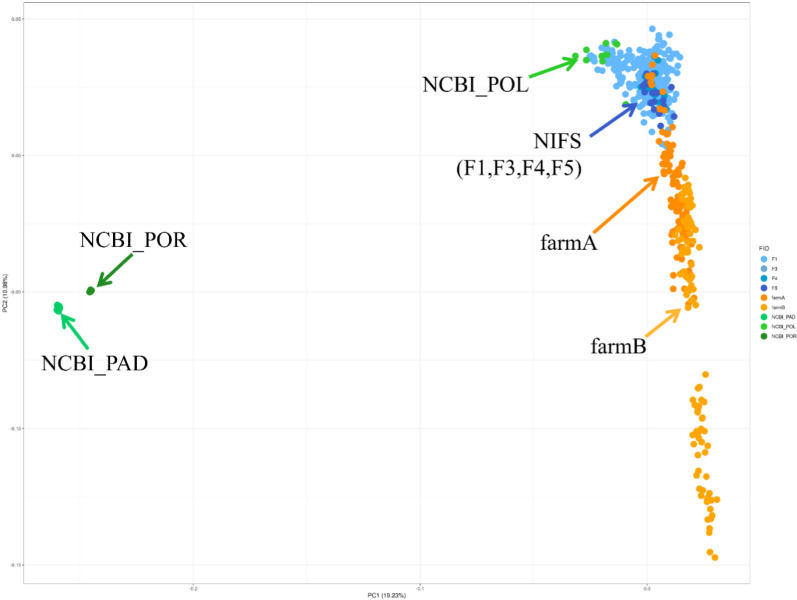
Principal component analysis. This PCA plot illustrates the genetic variation among different populations, with PC1 (19.23%) and PC2 (10.98%) explaining the majority of the variance. Each point represents an individual, and populations are color-coded as indicated in the legend. farmA (orange) and farmB (yellow) form distinct and separate clusters, highlighting their unique genetic profiles. NCBI_PAD (green) and NCBI_POR (deep green) cluster closely but remain distinct from other populations, while NCBI_POL (light green) and NIFS (blue tones) form tightly packed clusters, indicating genetic homogeneity within these groups.

Principal component analysis (PCA) was performed to examine the genetic structure among Korean olive flounder (Paralichthys olivaceus) populations (**Fig 2**). The cluster containing the groups *P. adspersus* (NCBI_PAD, green) and *P. orbignyanus* (NCBI_POR, deep green) from NCBI formed a tight cluster that was distinctly separated from the other populations along PC1 (19.23%), suggesting significant genetic differences. The NCBI *P. olivaceus* cluster (NCBI_POL, light green) was tightly grouped together, distancing with other groups. In farmed populations, the farm A (orange) and NIFS (blue tones) groups were located closely together and somewhat overlapping, indicating minimal genetic differentiation between them. The farm B (yellow) group, while close to the farm A and NIFS groups along PC1, was still distinguishable, suggesting it had moderate genetic differentiation from these groups. Along PC2 (10.98%), the farm B group showed a widespread distribution, indicating high genetic variability within the group along this component.

#### Phylogenetic tree (neighbor-joining tree) and Fst analysis.

**Fig 3:**
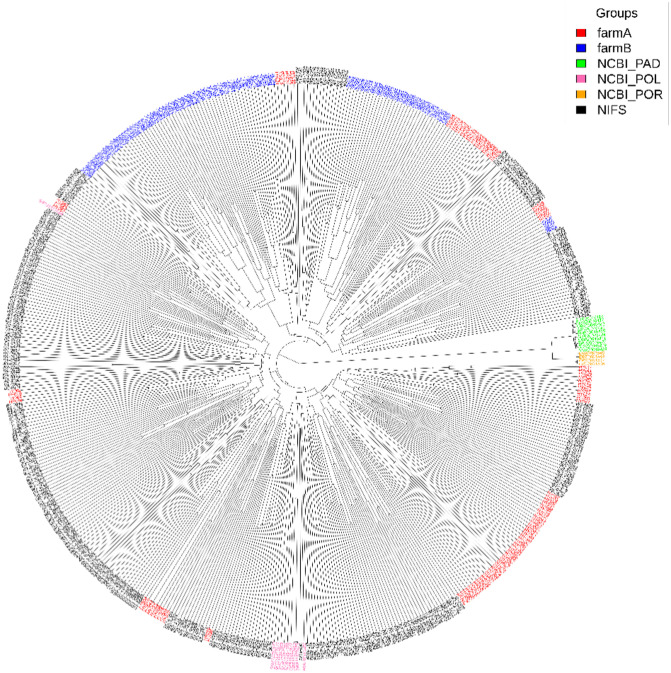
Phylogenetic tree of wild and farmed populations. The phylogenetic tree illustrates the genetic relationships among Korean olive flounder populations. NCBI_POL, representing the founder population, is genetically distant from other groups and occupies an independent position. NCBI_PAD and NCBI_POR exhibit similar genetic structures, while the NIFS population is positioned at the center of the tree, demonstrating close genetic connectivity with the farmed populations, FarmA and FarmB.

To examine genetic similarity and phylogenetic differentiation among populations, we performed phylogenetic tree and Fst analysis with 302, 200, and 24 animals for NIFS, aquaculture farms, and wild population (**Fig 3**). The tree had multiple main branches, each representing a distinct genetic cluster. Two species from the NCBI dataset, *P. adspersus* (NCBI_PAD, green) and *P. orbignyanus* (NCBI_POR, orange) formed a branch together before joining with the *P. olivaceus* (NCBI_POL, pink) branch, indicating a close relationship. The NIFS samples (black) were divided into two relatively distant clusters, as were the farm A samples (red). Each farm A cluster was closer to the respective NIFS cluster, reflecting a close genetic relationship between the NIFS and farm A datasets. The farm B group (blue) also formed two distinct clusters; however, one of these clusters was genetically closer to the farm A and NIFS groups, while the other cluster was more closely related to species in the NCBI dataset.

Fst analysis was conducted to evaluate the genetic structure and differentiation between Korean olive flounder populations (NIFS, FarmA, FarmB) and wild populations (NCBI) (Table 1). The results indicate that NIFS, FarmA, and FarmB are genetically very similar, suggesting potential gene flow during the breeding and aquaculture process. In contrast, high genetic differentiation was observed between NIFS and both NCBI_PAD (Fst = 0.2952) and NCBI_POR (Fst = 0.2743). Similarly, FarmA and FarmB also exhibited significant genetic differentiation from NCBI_PAD and NCBI_POR. Notably, the genetic differentiation between NCBI_PAD and NCBI_POR (Fst = 0.6791) was the highest, indicating minimal genetic similarity between the two groups. Overall, the results suggest strong genetic connectivity among NIFS and farmed populations, whereas NCBI populations maintain an independent genetic structure.

**Table 1:** Pairwise Fst values among korean olive flounder populations.

Population1	Population2	Fst
NIFS	farmA	0.021
NIFS	farmB	0.043
NIFS	NCBI_POL	0.041
NIFS	NCBI_PAD	0.295
NIFS	NCBI_POR	0.274
farmA	farmB	0.035
farmA	NCBI_POL	0.062
farmA	NCBI_PAD	0.314
farmA	NCBI_POR	0.288
farmB	NCBI_POL	0.099
farmB	NCBI_PAD	0.321
farmB	NCBI_POR	0.297
NCBI_POL	NCBI_PAD	0.423
NCBI_POL	NCBI_POR	0.337
NCBI_PAD	NCBI_POR	0.679

NIFS, FarmA, and FarmB populations exhibit very low genetic differentiation (Fst < 0.05), indicating close genetic relationships. High genetic differentiation (Fst > 0.27) is observed between NIFS and the wild populations (NCBI_PAD and NCBI_POR). The greatest differentiation is between NCBI_PAD and NCBI_POR (Fst = 0.679), showing minimal genetic similarity.

#### Admixture analysis.

**Fig 4:**
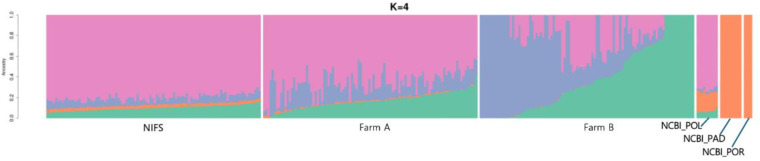
Admixture analysis of wild and farmed populations at K = 4. Each color represents a unique ancestral proportion. The x-axis denotes individual samples grouped by their respective populations; the y-axis indicates the proportion of genetic ancestry from each lineage.

Admixture analysis was conducted in this study to investigate the genetic ancestry and population structure of Korean olive flounder. This analysis assuming four ancestral populations (number of ancestry groups K = 4 in S2 Fig) revealed detailed insights into the genetic relationships among the species (Fig 4). In the ADMIXTURE analysis, the optimal number of ancestral populations (K) was determined using cross-validation (CV) error, a statistical metric employed to evaluate the predictive accuracy of the model for each K value. K = 4 was selected as the optimal value, representing a balance between model complexity and interpretability. The NIFS population, represented predominantly by one ancestral population (magenta), with a very small proportion of the second and third ancestral populations (blue and orange), exhibited a genetic structure that closely resembles the *P. olivaceus* population from NCBI (NCBI_POL), which is a wild population. The farm A population, also largely represented by magenta, showed a structure similar to NIFS. The farm B population, while also displaying a substantial magenta component, showed more genetic diversity compared to farm A. This increased diversity was indicated by the presence of additional green and blue components. In contrast, NIFS and farmed populations differed markedly from the wild species *P. adspersus* (NCBI_PAD) and *P. orbignyanus* (NCBI_POR). Those wild populations were characterized by distinct genetic structures, as evidenced by the prominent orange component.

**Fig 5:**
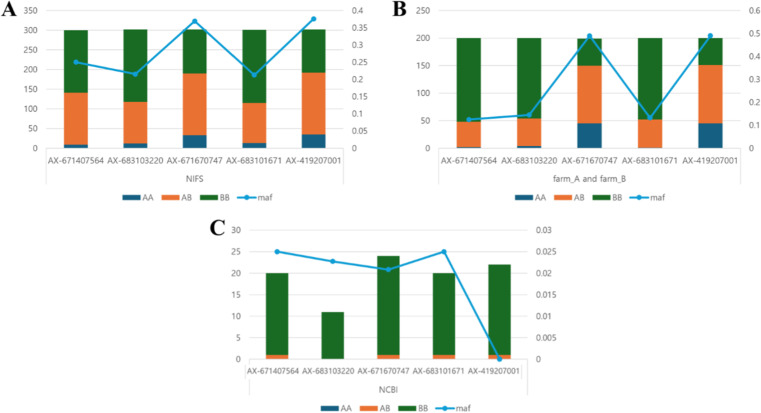
Comparison of SNP genotype distributions and minor allele frequencies (MAF) among NIFS, farmed, and wild Olive Flounder Populations. This is the result of checking the MAF of five SNPs that overlap across all three populations, selected from the significant SNPs identified in the GWAS for body weight at 18 months in the NIFS population.

This analysis was conducted to evaluate the genetic relationships and differences among the NIFS population, farmed populations (FarmA, FarmB), and wild populations (NCBI) by comparing the frequencies and distribution patterns of SNPs associated with body weight of 18 months (**Fig 5**). Specifically, the study aimed to assess the genetic influence of the NIFS core population on farmed populations by examining the similarity in SNP frequencies between these groups. The results present the minor allele frequency (MAF) and the distribution of genotypes (A, B, AB) for five significant SNPs identified in the GWAS of body weight at 18 months, which were commonly observed across NIFS, FarmA & FarmB, and NCBI populations. In the NIFS population, the MAF ranged between 0.35 and 0.4, indicating relatively high genetic diversity. Similarly, in FarmA and FarmB, the MAF ranged between 0.4 and 0.5, slightly higher than that of the NIFS population, reflecting a close genetic relationship between these farmed populations and NIFS. In contrast, the MAF in the NCBI wild population was consistently low across all SNPs, with some SNPs showing values below 0.02.

### Genetic evaluation

#### Estimated breeding values of three populations.

**Fig 6:**
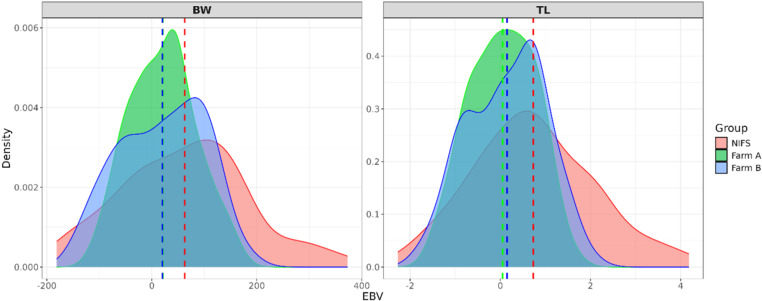
Comparison of average genomic breeding value of the 7^th^ generation of NIFS and two commercial farms. The genomic estimated breeding values (gEBV) for body weight (BW) and total length (TL) were compared across three populations: NIFS and two aquaculture farms (Fig 6) to assess the genetic levels of two farms based on NIFS. For BW, compared to NIFS, the mean values for farm A and B were similar, both being approximately zero. The distribution of farm B showed greater genetic diversity. For TL, compared to NIFS, the mean values for both aquaculture populations were lower, with the values of farm B being slightly higher than that of farm A.

The genomic estimated breeding values (gEBV) for body weight (BW) and total length (TL) were compared across three populations: NIFS and two aquaculture farms (Fig 6) to assess the genetic levels of two farms based on NIFS. For BW, compared to NIFS, the mean values for farm A and B were similar, both being approximately zero. The distribution of farm B showed greater genetic diversity. For TL, compared to NIFS, the mean values for both aquaculture populations were lower, with the values of farm B being slightly higher than that of farm A.

#### Prediction accuracy of estimated breeding value.

**Fig 7:**
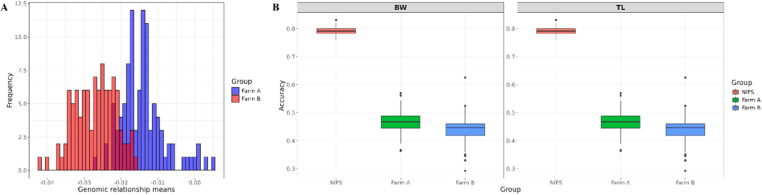
Comparison of genomic relatedness and prediction accuracy across the 7^th^ generation of NIFS and two commercial farms. The genetic relatedness between NIFS and farmed populations was compared (Fig 7-A). The genomic relationship means for the farm A primarily ranged from −0.027 to 0, while for the farm B group, they ranged from −0.042 to −0.016. Fig 7-B shows the accuracy of estimated breeding values (EBVs) for BW and TL across three populations: 7^th^ generation of NIFS (red), farm A (green), and farm B (blue). The highest mean accuracy, of 0.79 for both traits, was exhibited by the NIFS, indicating a high genetic relatedness with the reference population.

To examine the potential for genetic improvement in aquaculture populations, comparison on genomic breeding values was made between selected breeding populations and aquaculture farms. The genetic relatedness between NIFS and farmed populations was compared (Fig 7A). The genomic relationship means for the farm A primarily ranged from -0.027 to 0, while for the farm B group, they ranged from −0.042 to −0.016. The mean genomic relationship for the farm A was −0.014, with most individuals clustering around this value. The farm B showed a broader distribution with a mean of −0.027, suggesting greater genetic variability.

Fig 7B shows the accuracy of estimated breeding values (EBVs) for BW and TL across three populations: 7^th^ generation of NIFS (red), farm A (green), and farm B (blue). The highest mean accuracy, of 0.79 for both traits, was exhibited by the NIFS, indicating a high genetic relatedness with the reference population. A mean accuracy of 0.47 is shown by the farm A, which is lower than that of the NIFS but higher than that of the farm B. The lowest mean accuracy of 0.45 was shown by the farm B population, suggesting that this population had a lower genetic relatedness with the reference population.

## Discussion

Genetic diversity analyses have shown that Korean olive flounder (NIFS and two commercial farms) exhibited unique genetic patterns compared to wild populations (NCBI_POL, NCBI_PAD, NCBI_POR). These differences are primarily attributed to the distinct evolutionary histories and breeding practices in different regions. Korean olive flounder has been subjected to intensive selective breeding programs aimed at enhancing breeding objective trait such as growth. In contrast, wild populations may have been exposed to different selective pressures, resulting in divergent genetic patterns.

The unique genetic patterns in Korean olive flounder were shaped by selective breeding and historical gene flow. Principal Component Analysis (PCA) and ADMIXTURE results revealed that the wild populations, including *P. adspersus* and *P. orbignyanus*, were genetically distinct from the Korean farmed populations, emphasizing their genetic uniqueness. This difference was also observed in the study on genetic relatedness analysis using microsatellite markers of Kang, Noh [17] on Korean olive flounder, and on Japanese olive flounder populations in Shikano, Shimada [18]’s study. The core and farmed populations clustered closely together. The farm B population, while showing greater variability, reflecting more diverse genetic contributions, was still genetically similar to NIFS and farm A. This suggested a shared genetic breeding history between populations. Phylogenetic analysis and MAF plot further illustrated the close genetic relationships within the Korean olive flounder populations.

The NIFS and aquaculture farms exhibit comparable genotype distributions and MAF patterns, strongly indicating that NIFS serves as the genetic origin for the two farmed populations (**Fig 5**). The relatively low MAF observed in the NCBI population, when compared to the SNP diversity in NIFS and the farms, underscores the genetic divergence shaped by selective breeding and gene flow within the farmed populations.

The NIFS population served as the genetic source, with clear migration events indicating gene flow to two commercial farms. This gene flow underscored the important role of NIFS in shaping the genetic structure of these farmed populations. ADMIXTURE analysis complemented these findings by showing that the NIFS and farmed populations shared a predominant ancestral component, closely related to the wild *P. olivaceus* population. Due to the distribution of fertilized eggs and the farm’s own breeding practices (**Fig 1**), historical gene flow from wild populations into NIFS, and subsequently to farm A and B, highlighted the integration of genetic material from wild species that was shown by a small contribution of genetic material of those species to the core and farmed population in ADMIXTURE. The genetic analyses can demonstrate the transfer of genetic material between populations, as shown in previous studies [19,20].

Genomic prediction accuracy for any given trait is heavily dependent on the genetic relationship between reference and target populations [21,22]. In this study, we showed that the accuracy of farm A was higher than that of farm B due to the close genetic relationship between NIFS and farm A. In previous studies, Fraslin, Yáñez [23] reported that reducing the relatedness between the reference and target populations resulted in decreasing accuracy of genomic prediction for sea lice count and body weight in Atlantic salmon. Also, less related reference and target populations also tended to result in highly biased predictions. Some studies using rainbow trout and common carp showed that the accuracy sharply decreases as genetic relatedness becomes more distant [24,25].

As shown by the prediction accuracy, the genetic relatedness between farm A and NIFS is higher than that between farm B and NIFS, implying that farm A has a greater genetic contribution from NIFS compared to farm B. Nonetheless, the breeding value did not show a statistically significant difference between two farmed populations (**Fig 6 and 7**). The inability to see a difference in estimated breeding values between two farmed populations can be explained through several potential factors. Firstly, the farm B population might have benefited from additional genetic diversity introduced through gene flow from other high-performing populations or wild relatives, enhancing its overall genetic potential. Previous studies, likewise, observed considerable genetic similarities between wild and farmed populations, which suggests possible introgression and genetic movement between populations [26,27]. Secondly, the environmental factors and management practices unique to the farm B population might have contributed to better phenotypic performance and, consequently, higher breeding values. Thirdly, the selection pressures on the farm B population and a different selection history to the farm A population might also have an impact on its higher breeding values. Importantly, the genetic distance measures how different the genetic makeup of two populations is, but it does not directly correlate with breeding values. Breeding values indicate the genetic potential for desirable traits, which may not always align with genetic distance [28].

## Conclusions

This study examined the genetic structure and relationships among NIFS, farmed populations (FarmA and FarmB), and wild populations (NCBI) using SNPs associated with growth traits. The findings revealed high genetic similarity between NIFS and farmed populations, suggesting that NIFS serves as the genetic origin for FarmA and FarmB. This genetic relationship reflects effective gene flow facilitated by breeding programs and the distribution of fertilized eggs. In contrast, the NCBI wild populations showed low MAF values and the dominance of specific alleles, indicating significant genetic differentiation and reduced genetic diversity compared to farmed populations. These results highlight the role of selective breeding in shaping the genetic structure of farmed populations while emphasizing the genetic independence of wild populations. The study provides critical insights for future breeding programs to enhance growth traits while preserving genetic diversity in aquaculture.

## Supporting information

S1 Fig Diagram showing the method of measuring the body parts of olive flounder. (DOCX)

S1 Note Data generation. (DOCX)

S2 Note Estimation of genetic parameter. (DOCX)
