# Supplementary figures and images for: Genetic diversity of Olive flounder (Paralichthys olivaceus) and the impact of selective breeding on Korean populations
Source: PLoS One. 2025 Apr 16;20(4):e0318672. doi: 10.1371/journal.pone.0318672 (PMC12002499; doi:10.1371/journal.pone.0318672)

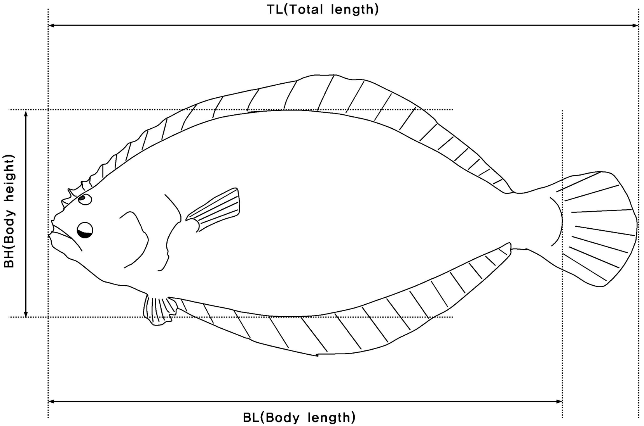


S1 Fig. Diagram showing the method of measuring the body parts of olive flounder

Supplement: S1 Fig — (DOCX) [file pone.0318672.s001.docx]
