# Supplementary material for: Genetic diversity of Olive flounder (Paralichthys olivaceus) and the impact of selective breeding on Korean populations
Source: PLoS One. 2025 Apr 16;20(4):e0318672. doi: 10.1371/journal.pone.0318672 (PMC12002499; doi:10.1371/journal.pone.0318672)
Supplement: S1 Note — (DOCX) [file pone.0318672.s002.docx]

S1 Note. Data generation

| Year | Number of Fertilized Eggs Distributed  (in thousands) | Broodstock Used for Fertilized Egg Production |
| --- | --- | --- |
| 2010 | 43,750 | First-generation offspring |
| 2011 | 65,510 | First- and second-generation offspring |
| 2012 | 77,900 | second-generation offspring |
| 2013 | 56,970 | second-generation offspring,  second-generation offspring of offsping |
| 2014 | 27,500 | second- and third- generation offspring,  first- and second-generation offspring of offsping |
| 2015 | 18,400 | third-generation offspring,  first-generation offspring of offsping,  third-generation offsping |
| 2016 | 50,570 | second-generation offspring,  third-generation offspring,  first-generation offspring of offsping |
| 2017 | 42,300 | fourth-generation offspring |
| 2018 | 39,300 | fourth-generation offspring |
| 2019 | 30,750 | fourth-generation offspring |
| 2020 | 6,900 | Fifth-generation offspring |

Between 2010 and 2020, over 450 million fertilized eggs were distributed to more than 70 aquaculture farms across the country. These eggs were derived from the selective breeding of superior broodstock across multiple generations (G1 to G5 in Fig. 1). During family production, 20,000 fertilized eggs were mixed and accommodated per 5-tonne tank (fibrereinforced plastic). On the 35th day after hatching, they were divided into 10,000 individuals (total length around 2 cm) per 5-tonne tank. On the 100th day after hatching, they were divided into 1,000 individuals (total length around 12 cm) per 20-tonne tank (concrete). On the 200th day after hatching, they were divided into 400 individuals (total length around 28 cm) per 20-tonne tank. From the 11th month after hatching to the end of production, 200 individuals (total length around 45 cm) per 20-tonne tank were divided, reared, and managed. A total of 992 fish belonging to 7 generations were used in this study.
