# Supplementary material for: Genetic diversity of Olive flounder (Paralichthys olivaceus) and the impact of selective breeding on Korean populations
Source: PLoS One. 2025 Apr 16;20(4):e0318672. doi: 10.1371/journal.pone.0318672 (PMC12002499; doi:10.1371/journal.pone.0318672)
Supplement: S2 Note — (DOCX) [file pone.0318672.s003.docx]

S2 Note. Estimation of genetic parameters

A total of 972 *Paralichthys olivaceus* from 1^st^ to 5^th^ generation of the NIFS dataset were used for the preliminary study. A linear mixed animal model using restricted maximum likelihood (REML) algorithm was applied to statistical analysis of body weight (BW) and total length (TL) using BLUPF90 program. The gBLUP model used is as follows:

$$\boldsymbol{y=Xb+Zu+ \varepsilon}$$

Where **y** is a vector of observed phenotypes, **X** is a design matrix relating the fixed effects to animals, **b** is a vector of fixed effects, **Z** is a design matrix which allocates the records in **y** to the random effects in **u** is a vector of random effects (estimated breeding values; EBVs), and **ε** is a vector of residual terms.

|  | **BW** | **TL** |
| --- | --- | --- |
| Genetic variance | 25721 | 0.243 |
| Residual variance | 15822 | 0.317 |
| Heritability | 0.69 | 0.43 |
